# Hemorrhagic Malarial Fever

**Published:** 1889-04

**Authors:** W. C. C. Stirling

**Affiliations:** Weaver, Texas


### HEMORRHAGIC MALARIAL FEVER.

BY W. C. C. STIRLING, M. D., WEAVER, TEXAS.

Malarial hemorrhagic fever is quite common in Texas on the creeks and rivers. Its distinguishing feature is hæmaturia. It has been called hæmaturic malarial fever, and is known in the malarial localities as black jaundice and swamp fever. A great many practitioners give it the name black jaundice, on account of the dark, black urine and yellowness of the skin and eyes.

The term hemorrhagic is to be preferred, as the hæmaturia is accompanied by hemorrhage in other situations. Malaria is not a hemorrhage inducing posion. The most experienced and accurate observers of malarial affections concur in the opinion that malaria never establishes the hemorrhagic diathesis; that it is only by changes effected in the human economy by its prolonged influence that it is capable of doing so.

The late Prof. S. M. Bemiss, of New Orleans, classes the morbid conditions of malarial fever with its tendency to hemorrhage as follows:

"First. The blood-changes of chronic malarial toxæmia so alter the consistency of that fluid as to favor the occurrence of hemorrhage. "Second. The long persistent states of malnutrition in chronic malarial cachexias produce textural weakening of the vascular walls and increased liability to their rupture." "Third. The increased blood-pressure put upon the vascular walls by passive congestion during a malarial paroxysm." The hemmorrhage generally takes place in the kidneys, but also takes place in other situations, viz., stomach, bowels, nose, gums and blistered surfaces. Sometimes the hemorrhage is thrown out into shut cavities as in parenchymatous structures.

I was called to see a lady on the 15th of September, that died on the 19th, from a hemorrhage on the brain. She was living in an intensely malarial locality, and had been suffering for several months with a malarial posion. She had pale, sallow complexion, legs anasarcous, pitting on pressure, enlarged spleen and liver.

On the morning of the 18th, she sank into a comatose condition, with head rolled to one side, with stertorous breathing, perspiring profusely with temperature 103°F. She was rational up to the morning of the 18th, but never spoke or moved after she sank into the coma. She had all the symptoms of apoplexy. She had a little girl that had hemorrhagic hæmaturia at the same time.

I had a case in August that the hemorrhage was from the nose and gums. This little patient came very near losing its life from the actual loss of blood. It is claimed by some of the best observers on malaria that the hemorrhage is not likely to endanger life. I have seen alarming hemorrhages from the extraction of teeth in patients suffering from chronic malarial posion. Yellowness of the skin and eyes always follow the hemorrhage. Observers differ as to the cause of the coloration. Some think it is the presence of bile-pigments, and others think its appearance is too rapid to ascribe it to obstruction. Professor S. M.

Bemiss thinks it is altogether improbable that it is due to sudden hypersecretion in such pathological states of the system as are present. He said, if, however, we account for it by saying that the addition of new toxic constituent, urea and its congeners, to an already profoundly poisoned fluid, suddenly arrests those processes which dispose of bile in physiological conditions of the system, it seems to me that we adopt the most rational theory." It is then jaundice from lack of consumption. Nausea, vomiting thirst and restlessness, are prominent symptoms. Delirium and coma are generally fatal symptoms. I never saw a case in either of those conditions that recovered. They generally die in a few hours. A short time ago I was sent for to see a young man that had a severe chill and hemorrhage of the kidneys at 2 p. m.; was delirious and died at 1 a. m.

All forms of the disease are dangerous, and should be treated promptly. The first important thing is to secure cinchona as early as possible. Louis & Lynch, Carroll's Prairie, Texas, claim that quinine sometimes produces the hemorrhage. They are old physicians, and have treated a great many cases. I can't agree with them. I have never seen hemorrhage follow the administration of quinine. I think in their cases the hemorrhage would have occurred if the quinine had not been given. All the works on malarial hemorrhagic fever that I have consulted, say that quinine should be given in large doses. Prof. S. M. Bemiss, who has practiced in a malarial locality for almost a half century, says he has never witnessed any symptoms following the administration of chincona salts which justified a belief that they increased the hemorrhage.

The next thing is to arrest the extravasation of blood, and to sustain the patient's strength. The best hemostatic we have is chloride of iron. It should be given every two hours in ten-drop doses. Ergot, gallic and sulphuric acid are valuable remedies to prevent the hemorrhage into the stroma of the kidneys and malpighian tufts. Bitartrate of potassium acts as a hemostatic by keeping up the flow of urine through the kidneys. For the jaundice we have no better remedy than calomel. It should be given at suitable intervals, until catharsis has been produced.

Milk, eggs, and milk-punch, should be given when necessary. Enfeebled action of the heart always calls for stimulants. In many cases the danger of the disease is manifested chiefly by the enfeebled action of the heart. Coldness of the surface is an indication for the external application of heat by means of warm blankets, hot mustard, foot bath, etc. Morphine and atropia should be given hypodermically for restlessness, delirium and convulsions. After the interruption of the paroxysm, quinine should be given in tonic doses, and tincture chloride of iron in twenty-drop doses after each meal, with a nutritious diet.

*Sequelea.*—A slow, imperfect convalescence not unfrequently follows a violent attack, attended with feeble digestion, muscular and nervous debility. Good tonics that contain quinine, and something to act on the liver is about all that is needed.

